# Microbial amelioration of salinity stress in HD 2967 wheat cultivar by up-regulating antioxidant defense

**DOI:** 10.1080/19420889.2021.1937839

**Published:** 2021-06-24

**Authors:** Madhulika Singh, Neha Tiwari

**Affiliations:** a Department of Botany, SSN College, University of Delhi, Delhi, India; b Department of Biotechnology, Delhi Technological University, Delhi, India

**Keywords:** Salinity, antioxidants, plant growth promoting bacteria, *P. indica*, abiotic stress

## Abstract

An experiment was conducted to investigate the potential of *Piriformospora indica* and plant growth-promoting bacteria (PGPB) to ameliorate salinity stress in HD 2967 wheat cultivar. Plants were treated with four different levels of salinity viz. 0, 50, 100 and 200 mM NaCl (electrical conductivity value 0.01, 5.84, 11.50 and 21.4 mS cm^−1^, respectively) under greenhouse conditions, using a completely randomized design experiment. Plants inoculated with PGPB and *P. indica* showed decrease in lipid peroxidation, relative membrane permeability and lipoxygenase enzyme (LOX) activity as compared to uninoculated plants. The result of this study showed that PGPB and *P. indica* inoculated HD 2967 wheat plants accumulated higher content of proline, α-tocopherol and carotenoid as compared to uninoculated plants. The HD 2967 wheat plants either inoculated with PGPB or *P. indica* showed significantly higher activities of antioxidant enzymes viz. superoxide dismutase, catalase and ascorbate peroxidase than that of the uninoculated plants. Moreover, PGPB inoculated plants showed greater activity of antioxidant enzymes than the plants inoculated with *P. indica*. Salinity stress tolerance was more pronounced in the PGPB inoculated than *P. indica* inoculated plants. This study revealed the potentiality of PGPB and *P. indica* as bio-ameliorator under salinity stress, and suggests that this plant microbial association could be a promising biotechnological tool to combat the deleterious effects of salinity stress.

## Introduction

As per an estimation of World Population Prospects (2020), the human population is currently increasing at the rate of 1.05% per year, and it is expected to be around 7.8 billion in 2021 Agriculture sector is facing a major problem to meet the food requirement of growing population. However, plant development and yield has been severely influenced by several abiotic stresses like salinity, drought, temperature, and heavy metals [1]. Among various abiotic stresses, salinity is one of major deleterious factors, which limits crop productivity, in various parts of world [2]. About 20% of cultivated land area is affected by salinity globally [3]. According to an estimate, about 50% of cultivated land will be affected by salinity by 2050, if salinization process continues at a current pace [4]. Agricultural land is affected by salinization primarily due to the excessive accumulation of soluble salts in the soil, particularly due to accumulation of NaCl salt [1].

Salinity stress often induces overproduction of reactive oxygen species (ROS) including hydroxyl radical (OH^•^), single oxygen (^1^O_2_), superoxide anion (O_2_^•―^) and hydrogen peroxide (H_2_O_2_) [5]. ROS formation causes oxidative damage to proteins, lipids and photosynthetic pigments [6]. ROS deteriorates the structure of cell membrane by oxidation of polysaturated fatty acids of the lipid layer, altering their permeability. However, plants have an excellent antioxidant system to ameliorate ROS induced oxidative stress via enzymatic and non-enzymatic antioxidant production [7,8]. Several studies have revealed that the enzymatic antioxidant system viz. ascorbate peroxidase (APX), catalase (CAT), superoxide dismutase (SOD), and the non-enzymatic antioxidants like as carotenoids, proline, α-tocopherol and ascorbic acid could combat oxidative stress and thereby facilitating plants to better adopt under saline conditions [7,8].

The level of lipid peroxidation largely increased under saline conditions [9], and has an influence on the plant membrane properties, protein degradation and the capacity of ion transport, and eventually triggers the process of plant cell death. However, ROS production under salt stress further initiates higher production of lipid peroxidation [10]. Excessive ROS production and peroxidation of lipids alters the activity of lipoxygenase enzymes [11], therefore enhance the conversion of lipids into hydroperoxyl fatty acids [12]. Salinity induced enhanced LOX activity subsequently led to cell death [11]. In turn, proline is an important scavenger of ROS, providing protection against oxidative damage under salinity stress [13] and APX imparts a defensive role against ROS [14]. Moreover, α-tocopherol scavenge free radicals responsible for oxidation of polyunsaturated fatty acids hence, protect cell membrane from lipid peroxidation and maintain membrane integrity in host plants [15]. Ascorbate is a major antioxidant molecule playing a crucial role in detoxification of ROS [16], protection of proteins and lipids during salinity stress [17]. Agami [18] reported that ascorbate application in barley improved plant growth, relative water content, proline, photosynthetic pigments, enzymatic antioxidants under salt stress. Under salinity stress, polyphenol accumulates in various plant tissues and scavenges free radicals [19]. Polyphenols regulate plant growth, pigment synthesis, scavenge ROS which help the plant to mitigate abiotic stresses viz. salinity, drought and temperature [20]. Carotenoids are the harvester of light during photosynthesis. Moreover, they act as chloroplast membrane stabilizers, hence reducing ion leakage and peroxidation of lipids [21,22]. The enzyme SOD catalyses the conversion of O_2_^.-^ to H_2_O_2_ [8] thus, plays a significant role in protecting plant from oxidative damage [23]. Catalase reduces the level of ROS by catalyzing the breakdown of hydrogen peroxide into water and oxygen [24].

In the recent years, several salt tolerant crops have been developed by conventional breeding program and recombinant DNA technologies, however, have been proven to be time consuming and cost intensive. In light of upcoming challenges, there is a need to develop alternative technologies for sustainable agriculture production, such as the use of soil microbes [25]. Several reports have revealed that the soil inoculation with beneficial soil microorganisms increases the production of antioxidant enzymes such as, SOD, CAT, and peroxidase (POD) which scavenge excess free radicals produced during salt stress [26], thus elevating salt tolerance in crop plants and increasing their growth. Amongst a wide array of such microorganisms, *P. indica* inoculation has been found to reduce salinity stress in rice plants [27] and drought stress in strawberry plants [28]. Further, *P. indica* colonization increases the activity of antioxidant defense enzymes in the plants exposed to salinity [29]. PGPB (*Bacillus cereus*) confer salinity tolerance in *Vigna radiata* by enhancing the activity of SOD, CAT and peroxidase which belongs to antioxidant defense system [30]. *Azotobacter chrococcum* alleviated salt stress in maize by increased accumulation of proline and polyphenols [31]. *P. indica* inoculation also enhances the accumulation of non-enzymatic antioxidants such as carotenoids and proline in rice plants [27] while grow under saline conditions. Recently, Kasotia et al. [32] reported that *Pseudomonas koreensis* colonization in soyabean plants stimulated the SOD and CAT enzyme activities under abiotic stress. The higher proline content has also been recorded in the plants colonized with arbuscular mycorrhizal fungi (AMF)[33].

The α-tocopherol a vitamin E compound located in the membrane of chloroplast and thylakoid and also in the plastoglobuli. This antioxidant deactivates ROS generated during photosynthesis (mainly ^1^O_2_ and OH^•^), and lowers rate of MDA production by scavenging lipid peroxyl radicals [34]. The α-tocopherol level changes in response to abiotic stress. High α-tocopherol content in microbial inoculated plants lowers lipid peroxidation [26] as the α-tocopherol involves in scavenging lipid peroxyl radicals. PGPB enhanced salinity tolerance in plants by increasing the production of non- enzymatic antioxidants such as α-tocopherol, ascorbate, carotenoids and polyphenols [35]. *P. indica* colonization in maize plant decreased the level of MDA production by stimulating antioxidant enzymes [36].

HD 2967 (Pusa Sindhu Ganga), a high yielding double-dwarf wheat variety, is one among the foremost necessary cereal crops in India. The plant growth and productivity are adversely affected by salinity. Till now, no systematic research has explored the effects of using *Azotobacter chroococcum, Enterobacter asburiae and Lactococcus lactis* in combination, in alleviating salinity stress in HD 2967 wheat cultivar. As per the literature survey, there is no report on *P. indica* induced salinity tolerance in the HD 2967 plant. Therefore, the present study was carried out with an aim to study the effect of *P. indica* and PGPB inoculation in HD 2967 wheat tolerance to salinity induced by NaCl treatment. It is hypothesized that *P. indica* and PGPB protect wheat plant from damaging effects of salt stress by enhancing antioxidant enzyme activities and antioxidant molecule accumulation.

## 2. Materials and methods

### 2.1. Experimental set up, growth conditions, and soil

A pot experiment was conducted under semi-control conditions (Temperature: 24–29°C; relative humidity: 60 ± 10%) in the botanical garden of Swami Shraddhanand College, University of Delhi, Delhi, India. Humidity was controlled by adequate spacing between plants, regular weeding, well-drained greenhouse floor, opening greenhouse door at night, installation of fans in greenhouse. The temperature was controlled by covering the green house by plastic sheets and installation of LED lights for uniform light distribution in greenhouse. Controlled humidity level discouraged the growth of pests. The soil used in the experiment was autoclaved to avoid contamination of soil.

The soil was collected from the fields of Narela Village, New Delhi, India after consulting from farmers. Six points in the fields were selected. Removed the vegetation, surface litter and 1 cm layer of soil. Soil was collected from a depth of 1–20 cm and placed in sacks and brought to green house. Soil was mixed and kept on a raised surface lined with plastic sheets. Soil was lightly pounded, sieved and air dried for 2 weeks. Soil was autoclaved at 121°C and 15 psi. The mechanical analysis of soil was done by sieve method and it was found that the percentage of very coarse particles was 2.84%, coarse sand 57.9%, fine sand 16.6% and silt and clay 22.66%. The physicochemical properties of the soil were determined by the department of soil sciences and agricultural chemistry, IARI, Delhi, India. The physicochemical properties of experimental soil are given in Table 1.

**Table 1.** Physicochemical properties of the experimental soil

Parameter	Properties/content
Soil type	Sandy loam (coarse particles: 2.84%, coarse sand: 57.9%, fine sand: 16.6% and silt and clay: 22.66%)
pH	7.5
Electrical conductivity	0.19 dS m^−1^
Organic matter	1.1%
Available N	196 mg kg^−1^
Available P	51.5 mg kg^−1^
Available K^+^	303 mg kg^−1^
Mg^2+^	245 mg kg^−1^
Zn ^2+^	7 mg kg^−1^
Fe^3+^	12.5 mg kg^−1^
Cu^2+^	4.04 mg kg^−1^
Mn^2+^	7.40 mg kg^−1^.
Na^+^	320 mg kg^−1^.

Before conducting an experiment, the soil was sterilized by autoclaving it for 30 minutes at 121°C and 15 psi to kill the prevailing microbes, if any. *Azotobacter chroococcum* is nitrogen solubilizer, *Enterobacter asburiae* is potassium solubilizer and *Lactococcus lactis* is phosphorus solubilizer. The experiment was laid out in a randomized complete block design with four factors: i) non-microbial control ii) inoculation with *P. indica* iii) inoculation with PGPB *(Azotobacter chroococcum, Enterobacter asburiae and Lactococcus lactis)* iv) four salinity levels (0, 50, 100 and 200 mM NaCl). Hence, there were 12 treatments (3 × 4) and each treatment had three replicates.

### 2.2. Plant material and inoculation treatment

Seeds of HD-2967 wheat cultivar used in the study, which were collected as a gift from the Department of Genetics, Indian Agricultural Research Institute (IARI), New Delhi, India. The seeds were surface sterilized by treating the it with 4% sodium hypochlorite for 1 minute and then washed three times with Milli Q water to remove any trace of chemical that could interfere with seed germination.

The sterilized seeds were directly sown in the earthenware pots containing 4 kg of autoclaved soil at a depth of 3 cm. Plants were watered twice a week. PGPB inoculum was obtained from the Department of Microbiology, IARI, New Delhi, India, and *P. indica* inoculum was collected as a gift from the Institute of Microbial Technology, Amity University, Noida, India. The microbes were added in the soil at the time of seed sowing at a depth of 3 cm. Same amount of sand was added in non-inoculated soil. Added recommended dose of fertilizer for pot experiment viz: nitrogen (N) as urea: 187.5 kg ha^−1^, diammonium phosphate (P): 75 kg ha^−1^, and muriate of potash as (K): 75 kg ha^−1^. One-third dose of nitrogen and complete doses of P and K were added in the soil at the beginning of experiment. The rest amount of nitrogen was equally divided and added after 21 d and 42 d of seed sowing, respectively.

### 2.3. Saline stress treatment

**Figure 1.**
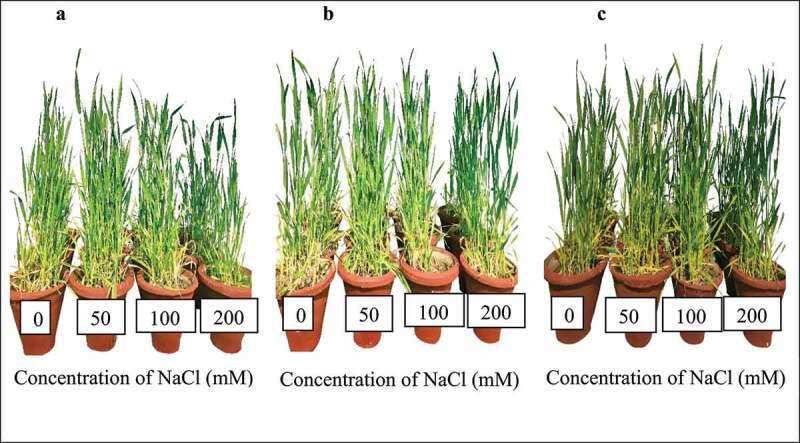
Experimental set-up showing (a) uninoculated wheat plants (control); (b) *P. indica* inoculated wheat plants; and (c) PGPBinoculated wheat plants after 80 d of sowing

Plants were watered with autoclaved tap water so that adequate moisture is maintained in the earthenware pots. Plants were allowed to grow for 20 d prior to the salt treatment to allow microbes to colonize. The plants were treated with four different levels of salinity stress (0, 50, 100 and 200 mM NaCl) once a week with 50 ml of each NaCl solution per pot for 60 d and a total of 400 ml of NaCl solution was added to each pot during the entire experiment. The electrical conductivity (EC) of soil was measured using conductivity meter (HACH analyzer, HQ440d multi) and it increased to 0.19, 6.03, 11.69, 21.59 mS cm^−1^ in the 0, 50, 100 and 200 mM NaCl salinity levels, respectively. The experiment was carried out under green house condition (light intensity: 1000 lux; temperature: 24–29°C and relative humidity: 60 ± 10). Plants were harvested by uprooting the entire plant manually after 80 d of sowing at stem extension second node visible seventh phenological stage of wheat development for analysis (Figure 1).

### 2.4. Root and shoot biomass estimation

Plants were harvested after 80 d of seed germination. Root and shoot samples were oven dried 2 d at 80°C to obtain constant weight and then root and shoot biomass was determined based on weights of the dried samples.

### 2.5. Oxidative damage estimation

The membrane relative permeability of leaves was determined following the protocol of Zwiazek and Blake [37]. Second leaf from top of the plants were rinsed with Milli Q water to eliminate electrolytes sticked to the surface of leaves. Leaf discs of 5 mm diameter were cut out. The leaf discs were submerged in test tubes containing 25 ml of Milli Q water. After incubation for one-hour, electrical conductivity of the solution was measured using conductivity meter (HACH analyzer, HQ440d multi). Thereafter, the leaf discs were boiled to allow lysis all cells. The solutions were cooled and conductivity was measured [37].

$$\begin{array}{r} Relative\;permeability \end{array}=\frac{EC\;of\;the\;solution\;before\;heating}{EC\;of\;the\;solution\;after\;heating}\times 100$$

The lipid peroxidation level in leaves was estimated in terms of malondialdehyde content (MDA), following the methods of Heath and Packer [38]. Leaf tissue (0.5 g) was crushed in 0.1% trichloroacetic acid (TCA). The homogenate was centrifuged at 12,000 rpm for 15 min at (Eppendorf, Centrifuge 5810 R). To the supernatant liquid 0.5% thiobarbituric acid (TBA) in 20% TCA was added. The mixture was heated and then cooled. The mixture was centrifuged for 10 min [38]. The absorbance reading of the supernatant was noted at 532 nm (Eppendorf UV-vis Spectrophotometer, BioSpectrometer basic model). The calculation of the MDA content was carried out using its extinction coefficient of 155 mM^−1^ cm^−1^ and expressed as n mole g^−1^ fw [38].

Lipoxygenase (LOX) activity was measured according to the protocol given by Doderer et al. [39]. Leaf tissue (500 mg) was crushed in phosphate buffer containing 0.5 mM EDTA. The homogenate was centrifuged at 12,000 rpm for 15 min. The enzyme activity was determined by adding 50 µL extract to 2.95 mL substrate, which was prepared by adding 70 µL of linoleic acid to 10 mL milli Q water containing 100 µL tween 20, final volume adjusted to 200 mL by using 0.1 M phosphate buffer solution [39]. Absorbance reading was noted at 234 nm and the activity of enzyme was expressed in µmol min^−1^g^−1^ fw.

### 2.6. Antioxidant molecules estimation

Leaf tissue (200 mg) was homogenized in 3% aqueous sulfosalicylic acid to determine the content of proline [40]. The homogenate was centrifuged at 12,000 rpm for 10 min. The essay mixture contained supernatant (2000 µL), acid ninhydrin, and anhydrous acetic acid. The mixture was boiled for 1 h and cooled immediately. Chromophore was extracted by adding toluene and absorbance was determined spectrophotometrically at the wavelength of 520 nm [40]. The proline content was determined from a standard curve and expressed in µmole g^−1^.

Leaf tissue (100 mg) was crushed in liquid nitrogen and homogenized in 0.1 N H_2_SO_4_ to determine the content of α-tocopherol [41]. The homogenate was centrifuged at 10,000 rpm for 10 min at room temperature. An equal amount of ethanol was added to the supernatant and centrifuged for 5 min. To the mixture, equal volume of xylene was added and centrifuged for 5 min. To 1 mL of upper phase, 0.12% (w/v) FeCl_3_ was added and incubated at room temperature [41]. The absorbance was observed at 520 nm wavelength. The content of α-tocopherol was expressed as µg g^−1^fw.

The content of carotenoids in plant leaves was estimated according to Hiscox and Isradtom [42]. Leaves (100 mg) were chopped into small pieces and it was dipped in 36 test tubes containing 7 ml dimethyl sulfoxide. The tubes were kept at 65°C for 4 h and final volume was made to 10 ml with dimethyl sulfoxide. The absorbance reading was recorded at 480 and 510 nm and the content of carotenoid was calculated as per the formula given by Arnon [43] and expressed as mg g^−1^fw.

### 2.7. Analysis of antioxidant enzymes

Leaf tissue (500 mg) was crushed in 0.1 M phosphate buffer (pH 7.5) solution containing 0.5 mM EDTA. The homogenate was centrifuged at 12,000 rpm for 20 min and the extract was used to measure enzyme activity.

SOD activity was determined following the protocol of Dhindsa et al. [44]. The reaction mixture contained 1.5 M sodium carbonate, 2 mM methionine, 2.25 mM nitro-blue tetrazolium, 3 mM EDTA, 100 mM potassium phosphate buffer, Milli Q water and 50 µL of enzyme. The reaction was started by adding 60 µM riboflavin and the test tubes were placed under 15 W fluorescent lamps for 15 min. The absorbance was read at 560 nm after the reaction was stopped [44]. The unit of enzyme activity was considered as the amount of enzyme in leaf tissue which decreased the absorbance to 50% as compared to the test tubes not having enzymes. The enzyme activity was expressed as unit min^−1^ g^−1^fw.

The CAT activity was determined following the protocol of Teranishi et al. [45]. The reaction mixture contains 6 mM H_2_O_2_, 0.1 M phosphate buffer (pH 7.0) and enzyme extract. The reaction was terminated using titanium reagent. The mixture was centrifuged at 10,000 rpm for 10 min and absorbance was noted at 415 nm [45]. The enzyme activity was expressed in unit min^−1^ g^−1^fw.

The APX activity was measured according to Nakano and Asada protocol [46]. To three mL of reaction mixture having potassium phosphate buffer (100 mM), ascorbic acid (3 mM), EDTA (3 mM), and H_2_O_2_ (6 mM), the enzyme extract was added for estimation of APX activity [46]. The change in absorbance was read at 290 nm and expressed as unit min^−1^ g^−1^ fw.

### 2.8. Statistical analysis

The data were analyzed using SPSS 21 statistical program (IBM SPSS Statistics 21) by one-way ANOVA with NaCl treatment, microbial inoculation and interactions among them as a source of variation. Comparison of the means was determined by post hoc Duncan’s test (p < 0.05).

## 3. Results

### 3.1. Shoot and root biomass

**Figure 2.**
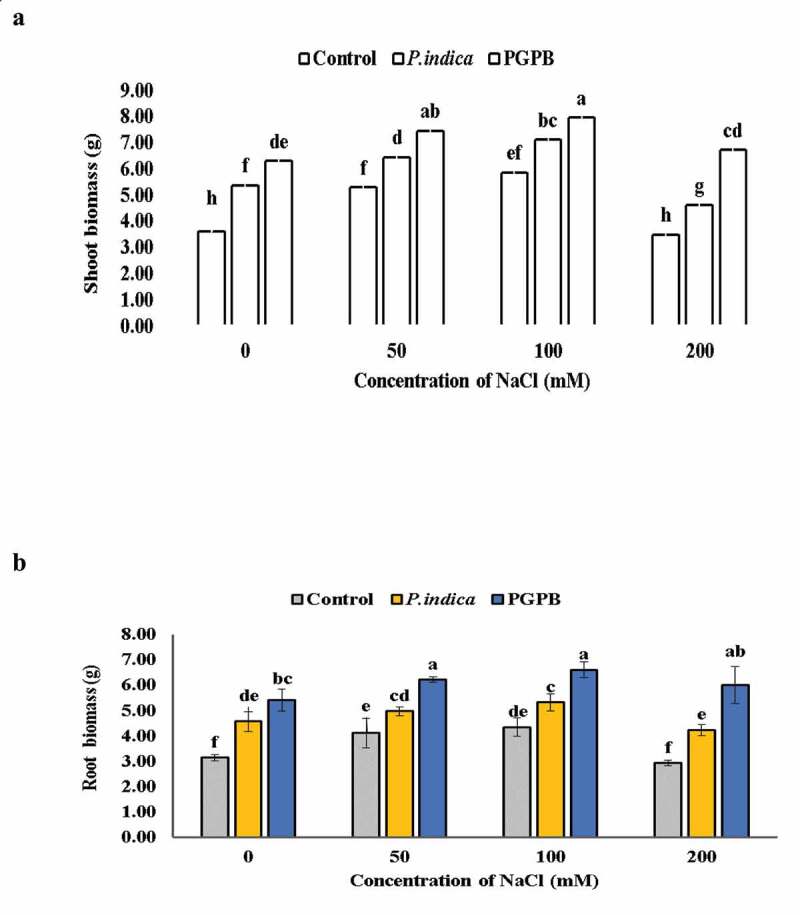
Effects of NaCl and microbial inoculation in HD 2967 wheat cultivar on (a) shoot biomass and; (b) root biomass. Values represent means ± S.D. (n = 3). Different letters indicate statistically significant differences for p < 0.05

Microbial inoculation of HD 2967 wheat variety showed higher plant growth at all levels of salinity as compared to the uninoculated plants. However, NaCl treatment (200 mM) lowered the shoot biomass by 3.6% compared to plants without NaCl treatments in uninoculated plants. The root biomass remained unchanged at 0 mM NaCl treatment as well as 200 mM NaCl treatment in uninoculated control plants, and decreased proportionally in case of *P. indica* inoculation. Root biomass appeared same at 50 and 100 mM NaCl, and a very diminutive difference was detected at 200 mM NaCl treatment in case of PGPB inoculation. On comparing the potential of PGPB and *P. indica* it was observed that PGPB inoculation exhibited a higher shoot and root biomass than *P. indica* colonized plants. At 200 mM NaCl salinity level, PGPB inoculation increased the shoot and root biomass by 48% and 51.25%, respectively, while *P. indica* inoculation increased it by a 24.4% and 30.89%, respectively, compared to un-inoculated plants (Figure 2 **a-**b). Biomass of shoot increased with increasing levels of salinity (0, 50 and 100 mM NaCl) with microbial inoculation; however, biomass decreased when wheat plants were supplied with 200 mM NaCl solution in both microbial inoculated and uninoculated plants. The dry weight was significantly greater in PGPB and *P. indica* inoculated under nonsaline conditions in comparison to uninoculated plants.

### 3.2. Oxidative damage

**Figure 3.**
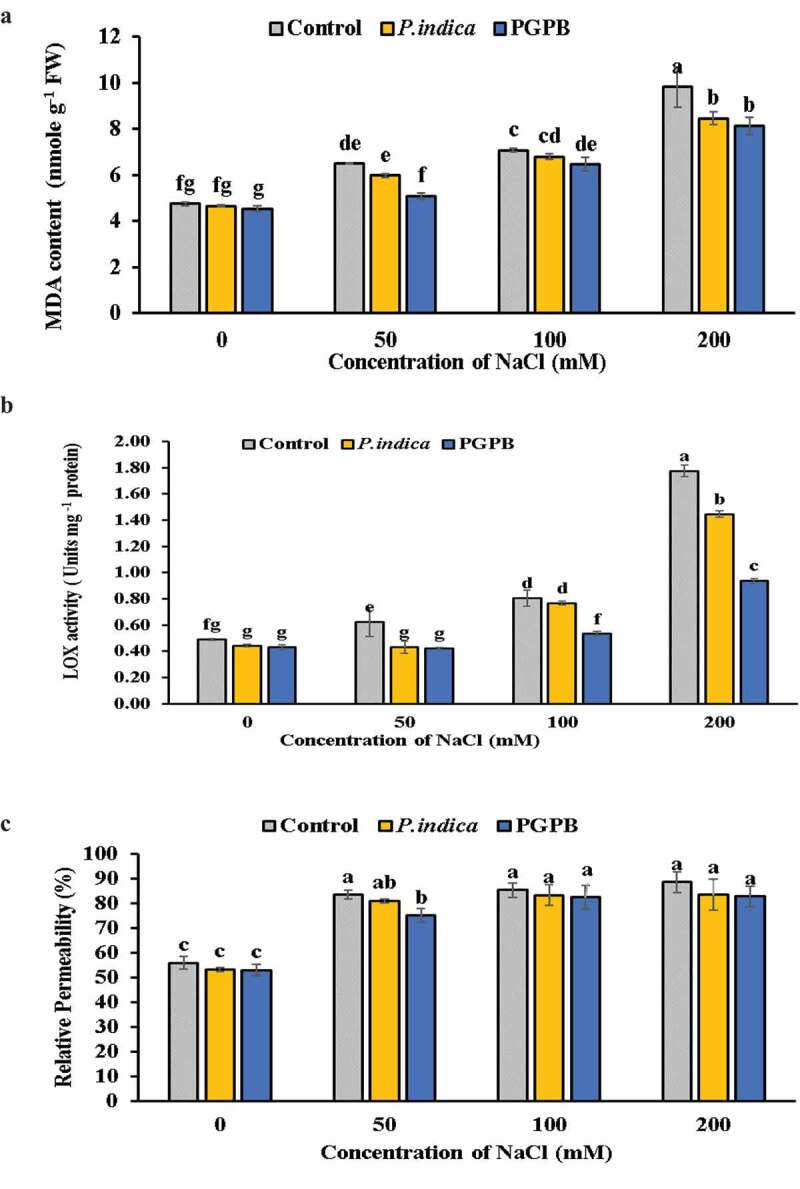
Effects of NaCl and microbial inoculation in HD 2967 wheat cultivar on (a) malondialdehyde content; (b) LOX activity and; (c) relative permeability. Values represent means ± S.D. (n = 3). Different letters indicate statistically significant differences for p < 0.05

Under saline conditions, MDA content in the leaves showed a rectilinear increase with increasing levels of salinity in microbial inoculated and uninoculated HD 2967 wheat cultivar. On the whole, PGPB and *P. indica* inoculated plants showed lower level of MDA than uninoculated control plants at each levels of salinity (Figure 3 a). Noteworthy was that there was no noticeable significant difference in lipid peroxidation level between response of PGPB and *P. indica* at 0 and 200 mM NaCl treatments.

LOX activity in the leaves of microbial inoculated and uninoculated plants increased with increased level of salinity; however, a significant difference was recorded between microbial inoculated and uninoculated plants (Figure 3 b). LOX activity in microbial inoculated plants was notably lower at each salinity level. However, PGPB inoculated plants showed lesser activity of LOX than that of the *P. indica*, at all levels of salinity.

As the levels of salinity increased, there was a gradual increase in the relative permeability of cell membrane in microbial inoculated and uninoculated wheat plants (Figure 3 c). There was no significant difference in relative permeability of membrane between *P. indica* inoculated plants and uninoculated plants at 0, 50 and 100 mM NaCl treatments; however, at 200 mM NaCl *P. indica* inoculated plants showed lower relative permeability of membrane than uninoculated plants. At 0 mM NaCl treatment PGPB inoculated plants showed no significant difference in relative permeability of membrane as compared to uninoculated plants; however, at 50, 100 and 200 mM NaCl treatments the relative permeability was significantly lower in PGPB inoculated plants as compared to uninoculated plants. There was no significant difference in relative permeability of membrane in PGPB and *P. indica* inoculated plants at 0, 100 and 200 mM NaCl treatments; however, at 50 mM NaCl PGPB inoculated plants showed lower relative permeability of membrane than *P. indica* inoculated plants.

### 3.3. Antioxidant molecules

**Figure 4.**
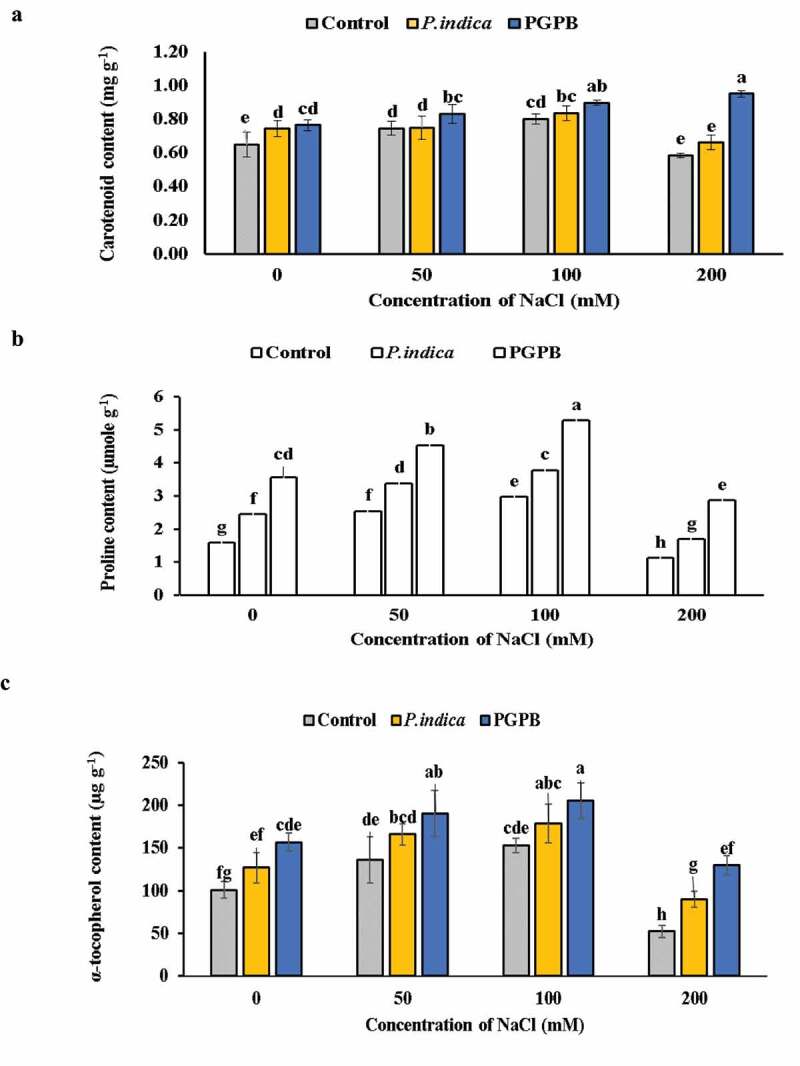
Effects of NaCl and microbial inoculation in HD 2967 wheat cultivar on content of (a) carotenoids; (b) proline and; (c) α-tocopherol. Values represent means ± S.D. (n = 3). Different letters indicate statistically significant differences for p < 0.05

PGPB and *P. indica* inoculation enhanced the antioxidant molecules content viz. carotenoids, α-tocopherol, and proline in HD 2967 wheat plant. The carotenoid content remained unchanged at 0 and 200 mM NaCl treatments in uninoculated control plants and there was no significant difference in carotenoid content at 50 and 100 mM NaCl treatments. There was no difference in the carotenoid content between *P. indica* inoculated and uninoculated plants at 50, 100, 200 mM NaCl salinity levels. The carotenoid content was remarkably higher in PGPB inoculated plants as compared to uninoculated plants at all levels of salinity. At 0 and 100 mM NaCl treatment there was no difference in carotenoid content between PGPB and *P. indica* inoculated plants. The content of proline and α-tocopherol was higher in microbial inoculated than uninoculated plants at all salinity levels (Figure 4 **a-**c). Accumulation of carotenoids, proline and α-tocopherol significantly increased with increasing levels of salinity (0, 50 and 100 mM NaCl); however, their content decreased when wheat plants subjected to 200 mM NaCl stress in both microbial inoculated and uninoculated plants. However, the accumulation of carotenoids and α-tocopherol was significantly greater in both PGPB and *P. indica* inoculated plants even when not subjected to salinity stress as compared to uninoculated plants.

### 3.4. Antioxidant enzyme activity

**Figure 5.**
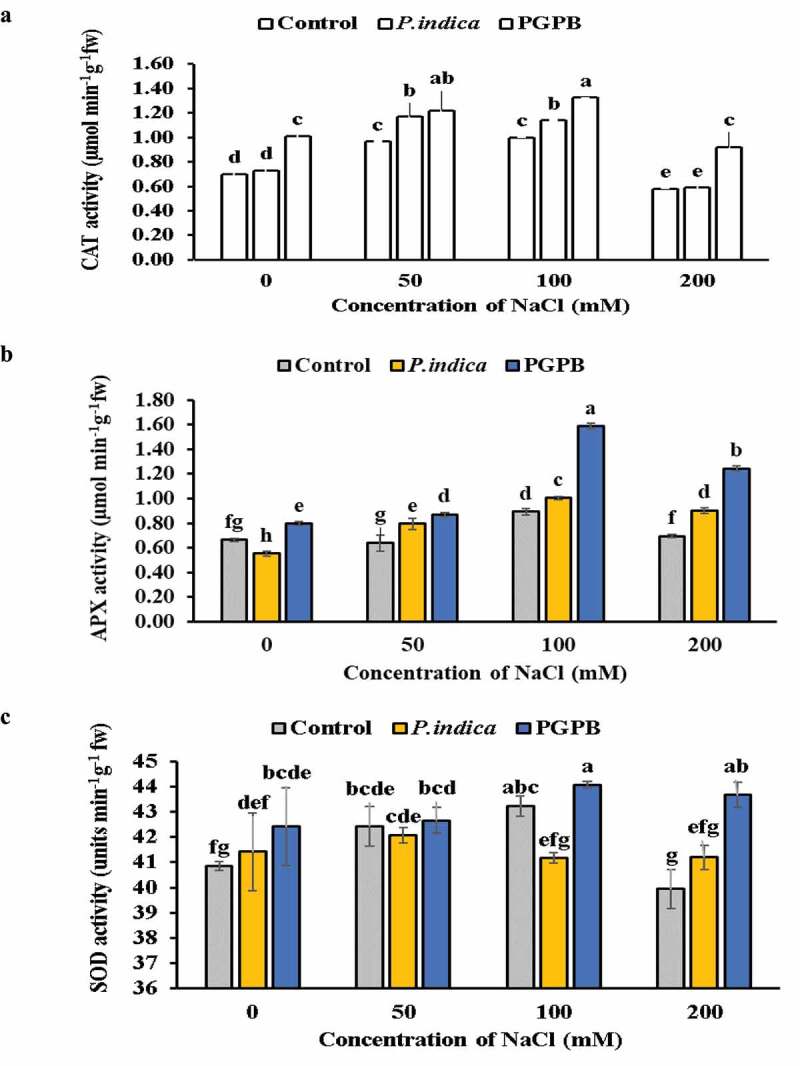
Effects of NaCl and microbial inoculated in HD 2967 wheat cultivar on activity of (a) CAT; (b) APX and; (c) SOD. Values represent means ± S.D. (n = 3). Different letters indicate statistically significant differences for p < 0.05

The activities of antioxidant enzymes (SOD, CAT and APX) were evaluated in microbial inoculated and uninoculated HD 2967 wheat plants at different levels of salinity (Figure 5 **a-**c). The activity of CAT and APX was higher in plants under salinity stress when inoculated with PGPB and *P. indica* as compared to uninoculated plants (Figure 5 **a-**b). Although microbial inoculated and uninoculated plant showed a significant increase in CAT and APX with increasing levels of NaCl (0, 50, and 100 mM NaCl); however, at 200 mM NaCl level their activity is declined. The SOD activity remained unchanged at 0 and 200 mM NaCl treatments in uninoculated control plants and there was no significant difference in SOD activity at 50 and 100 mM NaCl salinity level in uninoculated plants. In *P. indica* inoculated plants SOD activity decreased with increase of salinity level at the activity remained same at 0 and 50 mM NaCl treatments and 100 and 200 mM NaCl treatments. In PGPB inoculated plants there was high SOD activity at 0, 100 and 200 mM NaCl salinity level as compared to uninoculated plants; however, activity remained same at 50 mm NaCl salinity level. The SOD activity was higher in PGPB inoculated plants as compared to *P. indica* inoculated plants at all levels of salinity.

## 4. Discussion

The crop plants subjected to salinity stress have deleterious effects on various biological processes via generation of ROS and thereby reduce plant growth, productivity and yield. Excessive production of ROS damages various cellular components such as nucleic acid, lipid and proteins, impacting the cellular integrity, which could lead to cell death. Salt stress has a deleterious effect on plant biomass production, enhance the production of ROS, and increase ion toxicity that limits plant cell functioning [47]. Soil microorganisms can be explored as plant growth stimulators and to minimizing deleterious effect of salinity via modulation of enzymatic and non-enzymatic antioxidant molecule accumulation. The present study demonstrated the efficacy of PGPB and *P. indica* in ameliorating salinity-induced oxidative damage in HD 2967 wheat cultivar via production of enzymatic and non-enzymatic antioxidant molecules. Findings in the present study showed a substantial increase in the plant growth as a result of PGPB and *P. indica* inoculation. Enhanced plant growth and yield by PGPB in wheat and *P. indica* in rice have also been previously reported [27,48].

Our results showed that shoot and root biomass was significantly reduced at 200 mM NaCl treatment in both microbial inoculated and uninoculated plants. The decrease in shoot and root dry weight was reported in maize varieties subjected to salt stress [49], which might be due to the production of ROS and increased ion toxicity [47]. The reduced biomass might be due to decreased accumulation non-enzymatic antioxidants (proline, carotenoids and α-tocopherol), decreased activity of enzymatic antioxidants (CAT, APX and SOD) and increased relative permeability, MDA content and LOX activity as reported in this investigation. However, higher shoot and root dry weight in PGPB and *P. indica* inoculated plants shows the positive effect of microbes on tolerance of HD 2967 under salinity stress. The effect of *P. indica* inoculation on enhanced biomass production has also been reported in barley [30], *Arabidopsis* [50] and maize [51] under salt stress. Microbial inoculation displayed an increase in dry weight of shoot and root at different levels of salinity which might be due to the increased accumulation of osmolytes, carotenoids, reduced ion leakage. These results are in accordance with the earlier findings of inoculation of PGPR in wheat [52] and *Zea mays* [53].

Salinity leads to production of excessive free radicals responsible for oxidative damage, and reduced plant growth [54,55]. Further, the overproduction of ROS leads to oxidation of polysaturated fatty acids of the membrane lipid layer, which is more likely under saline conditions. The MDA, which is a product of peroxidation of lipids, is considered as an indicator of oxidative damage induced by salt stress. Lipid peroxidation largely influence the ultrastructure of cellular membrane, alter their permeability, and inactivate membrane-linked receptors [10]. This study revealed that salinity-induced ROS generation leads to the higher level of MDA content in both microbial inoculated and uninoculated plants at all salinity levels. Increased LOX activity also increased lipid peroxidation level in plants as reported in this study. Plants subjected to 200 mM NaCl accrued more MDA content and membrane damage. This observation supports previous findings which showed that salinity stress induces enhanced production of MDA [56], thereby affecting the integrity of membrane [57]. However, microbial inoculation showed lower MDA content and relative permeability of membrane (Figure 3 c) than uninoculated plants at all levels of salinity, which indicates that PGPB and *P. indica* inoculation reduces oxidative damage and maintains the integrity of cellular membrane. The PGPB and *P. indica* were able to maintain lower MDA content by activating the adaptive responses such as synthesis of non-enzymatic molecules (carotenoids, proline and α-tocopherol) and enzymatic antioxidants (CAT, APX and SOD). These findings are similar to previous findings on improved water stress tolerance of *P. indica* inoculated rice by maintaining low MDA content through enhanced ROS scavenging system [58]. This observation is in accordance with earlier report which shows that *Bacillus aquimaris* inoculation in *Zea mays* [59] and AMF inoculation in tomato [60] reduced the level of lipid peroxidation and ion leakage.

There was a remarkable difference in membrane leakage between inoculated and uninoculated plants. The inoculated plants had lower leakage as compared to uninoculated plants. Thereby it shows that wheat plants on inoculation could have better management of cellular membrane integrity and its permeability. Furthermore, lower amount of MDA content, LOX activity and higher content of carotenoids, proline and α-tocopherol in inoculated plants confirms that microbial inoculation provides better safeguard under salt stress conditions. These results support previous observation of such lower MDA level and LOX activity in *Brevibacterium linens* inoculated *Oryza sativa* plant subjected to salinity stress [61]. Waller et al. [29] reported that there was enhanced production of antioxidants in *P. indica* inoculated barley, which reduces the salinity-induced lipid peroxidation. However, these findings do not corroborate the previous findings of Kumari et al. [62] where there was no correlation between lipid peroxidation level and LOX activity in bacterial colonized soyabean plants under salt stress.

LOX enzyme converts lipids into hydroperoxyl fatty acids [12]. Salinity induced enhanced LOX activity is linked with the production of free radicals and peroxidation of lipids, causing damage to the cell. LOX activity showed a rectilinear increase with the increase in salinity level in microbial inoculated and uninoculated plants. However, compared to uninoculated, LOX activity in microbial inoculated plants was notably lower at each level of NaCl treatment, thus reducing lipid peroxidation, maintaining membrane integrity, reducing ion leakage and enhanced plant growth. The PGPB and *P. indica* were able to maintain lower LOX activity by activating the synthesis of non-enzymatic molecules (carotenoids, proline and α-tocopherol) and enzymatic antioxidants (CAT, APX and SOD). The reduced LOX activity in microbial inoculated plants is in agreement with earlier report in rice plant [61].

The non-enzymatic antioxidants include carotenoids and proline [63]. In the present study, salinity stress led to enhanced accumulation of antioxidant molecules viz. carotenoids, proline and α-tocopherol in inoculated as compared to uninoculated plants thereby showing a better management of MDA content and cellular membrane integrity. These results support previous observation of such high accumulation of antioxidant molecules in fenugreek plants grown in a saline soil [33]. Lower level of lipid peroxidation in microbial inoculated plants could be a result of high content of α-tocopherol synthesis, which disrupts lipid oxidation pathway [64].

Carotenoids are the harvester of light during photosynthesis. Moreover, they act as chloroplast membrane stabilizers, hence reducing ion leakage and peroxidation of lipids [21,22]. In the present study, the increased accumulation of carotenoids in PGPB and *P. indica* inoculated plants lowered the oxidative damage, as carotenoids prevent generation of singlet oxygen [65]. Proline has a major role in osmotic adjustment, stabilizing subcellular structures, and scavenge ROS under drought stress [13]. Indeed, proline accumulation under water and salinity stress may facilitate plants to withstand such environmental stresses. The increased content of proline in PGPB and *P. indica* colonized wheat plants indicated greater salt tolerance as compared to uninoculated plants which might be due to reduced lipid peroxidation and increased membrane stability, signifying a role of proline as an indicator for salinity tolerance [25,66]. The increased proline content has also been found to alleviate oxidative stress caused by salt stress in microbial inoculated maize plants [67], *P. indica* inoculated rice plants [28] and in PGPR inoculated soybean [68].

The α-tocopherol being an antioxidant deactivates ROS generation during photosynthesis (mainly ^1^O_2_ and OH^•^) and lowers lipid peroxidation level by scavenging lipid peroxyl radicals [34]. High content of α-tocopherol in PGPB and *P. indica* colonized plants as compared to microbial uninoculated plants confirms the role of α-tocopherol as free radical scavenger and lipid peroxidation modulator in plants during salinity stress [26,64]. These results substantiate the fact that higher content of α-tocopherol in AMF colonized fenugreek plant mitigates injurious effect of salt stress as has been reported by Evelin and Kapoor [33]. Present study revealed that inoculation of wheat plants by PGPB and *P. indica* enhanced salt tolerance in wheat via regulating cellular enzymatic and non-enzymatic antioxidant system.

There was a remarkable difference between antioxidant enzyme activity of microbial inoculated and uninoculated plants. At each level of salinity, microbial inoculation exhibited better plant growth than uninoculated plants, which may be due to increased activity of ROS scavenging antioxidant enzymes (CAT, APX and SOD). Both PGPB and *P. indica* inoculated plant exhibited higher activities of SOD as compared to uninoculated plants under salinity stress. SOD has a significant role in protecting plant from oxidative damage by converting O_2_^.-^ to H_2_O_2_. H_2_O_2_ generated needs to be detoxified by conversion to H_2_O. Further, the speedy scavenging of H_2_O_2_ is done by enhanced activities of CAT and APX, which catalyze the breakdown of H_2_O_2_ produced by SOD thus protecting the cell from oxidative damage. At all salinity level, the CAT and APX activities were more in microbial inoculated plants than uninoculated plants. CAT and APX activity increased with increasing salinity levels (0, 50 and 100 mM NaCl), however, their activity declined at 200 mM NaCl treatment. This may be attributed due to following factors: (i) salinity stress aggravates CAT destruction by proteinases, (ii) decrease iron content under salinity stress [69,70], and (iii) synthesis of new enzymes are prevented by salinity stress [71]. Overall, the present study exhibited lower oxidative stress in PGPB and *P. indica* that may be a result of (i) improved membrane stability by lowered lipid peroxidation, loss of ions and LOX enzyme activity, and (ii) a better antioxidative capacity of PGPB and *P. indica* inoculated than uninoculated plants.

In conclusion, PGPB and *P. indica* inoculation enhanced the production of non-enzymatic antioxidant molecules and activities of antioxidant enzymes at all levels of salinity. Therefore, PGPB and *P. indica* inoculated HD 2967 wheat cultivar possessed better antioxidant defense system to prevent oxidative damage, and therefore more tolerant to salinity stress. The results presented in this study clearly indicated that PGPB and *P. indica* improved salt stress tolerance potential of HD 2967 wheat plants, by down regulating the ROS response due to improved enzymatic and non-enzymatic antioxidant system. The enhanced level of antioxidant molecules in PGPB and *P. indica* inoculated plants under salinity stress, also substantiate the supposition that plant–microbe interaction could ameliorate salinity stress and tolerance to salt stress in HD 2967 wheat cultivar.
